# Supplementary material for: Explaining subscription intention for video streaming platforms in China: Integrating the UTAUT2 model, perceived value theory, and S-O-R theory
Source: PLoS One. 2025 May 8;20(5):e0322860. doi: 10.1371/journal.pone.0322860 (PMC12061175; doi:10.1371/journal.pone.0322860)
Supplement: S1 — (DOCX) [file pone.0322860.s001.docx]

Inclusivity in global research

PLOS’ policy on inclusivity in global research aims to improve transparency in the reporting of research performed outside of researchers’ own country or community and ensures that PLOS publications reporting global research adhere to high standards for research ethics and authorship. Authors of relevant research articles may be asked to complete the questionnaire below, which outlines ethical, cultural, and scientific considerations specific to inclusivity in global research. This questionnaire may be requested when researchers have travelled to a different country to conduct research, if research uses samples collected in another country, research with Indigenous populations or their lands, or if research is on cultural artefacts. Researchers travelling to another country solely to use laboratory equipment will not normally be required to complete the questionnaire. However, the questionnaire can be requested at the journal’s discretion for any submission – if you have been requested to complete this questionnaire by the PLOS journal you submitted to, please do so.

Please complete the questionnaire below and include this as a Supporting Information file with your manuscript. Note that if your paper is accepted for publication, this checklist will be published with your article in the supporting information files. Please ensure that you reference the checklist in the main body of your manuscript. We suggest adding a subsection ‘Inclusivity in global research’ to your Methods section and adding the following sentence: “Additional information regarding the ethical, cultural, and scientific considerations specific to inclusivity in global research is included in the Supporting Information (SX Checklist)”

The questions have been designed to be applicable to a wide range of study types, and there are subsections for both human subjects research and non-human subjects research. If any of the questions are not relevant to your research please mark them as “N/A” as appropriate.

**Ethical considerations, permits and authorship**

*This section is applicable to all research types.*

Provide details as to who granted permissions and/or consent for the study to take place in the Methods section of your manuscript. This should include the names of **all** ethics boards, governmental organizations, community leaders or other bodies that provided approval for the study. If individuals provided approval refer to these people by their role or title but do not list their name(s).

As detailed on page 11, line 11, in the "Inclusivity in Global Research" section of the Methodology: ‘Additional information regarding the ethical, cultural, and scientific considerations specific to inclusivity in global research is included in the Supporting Information (Appendix 1 Checklist). This study was granted approval by the Human Ethics Committee (HEC), School of Communication, Qingdao University of Science and Technology, China.’

If there were any deviations from the study protocol after approval was obtained please provide details of these changes in the Methods section of your manuscript.

There is no deviations from the study protocol occurred after obtaining ethical approval.

Did this study involve local collaborators that are residents of the country where the research was conducted or members of the community studied? If you do not have any authors from said communities, please provide an explanation for this below.

Everyone listed as an author should meet PLOS’ criteria for authorship and all individuals who meet these criteria should be included in the author byline, rather than the acknowledgements. For further information please see the journal’s Authorship Policy.

While this study did not require participants to belong to any specific community, Prof. Chen (corresponding author, China citizen) and Dr. Wu Tong (first author, China citizen) played an active roles in data collection among potential local subscribers of online video streaming services in China. All contributors meeting PLOS’ authorship criteria are recognized in the author byline.

**Human subjects research (e.g. health research, medical research, cross-cultural psychology)**

Did you obtain written informed consent from a representative of the local community or region before the research took place? How did you establish who speaks for the community? Details of written informed consent obtained from study participants should be reported separately in the Methods section of your manuscript.

As demonstrated by the written informed consent included on the cover page of the online questionnaire, this study complies with ethical standards by ensuring that all participants were thoroughly informed about the nature and purpose of the research.

For detailed information regarding the written informed consent, refer to Page 11, line 5, Section Methodology of the manuscript.

How did members of the local community provide input on the aims of the research investigation, its methodology, and its anticipated outcome(s)?

When engaging with the local community, how did you ensure that the informed consent documents and other materials could be understood by local stakeholders?

The respondents in this study are potential subscribers to online video streaming platform and are not limited to any specific community. Any Chinese adult who meets the eligibility criteria (see Section Methodology in the manuscript, page 9) could be invited to participate and provide input through the online survey. Participants maintained full autonomy to withdraw from the survey at any time, ensuring their right to privacy. Stringent measures were taken to protect the confidentiality of participant data, in full compliance with the Personal Data Protection Act. The study received ethical approval from the Human Ethics Committee (HEC) at the authors’ university in China.

Will the findings of the research be made available in an understandable format to stakeholders in the community where the study was conducted (e.g. via a presentation, summary report, copies of publications, etc.)? Please provide details of how this will be achieved.

This study complies with ethical standards by ensuring that all participants were fully informed about aims and nature of the research, evidenced by the written informed consent provided on the cover page of the online distributed survey.

**Non-human subjects research using specimens/ animals collected as part of the study, or those housed in archival collections. Examples include archaeology, paleontology, botany and zoology.**

Did the permission you obtained from a local authority to perform the study include an agreement on access to outputs and benefit sharing? This may include procedures to enable fair distribution of the benefits and resources arising from the research performed. Please include any details of Prior Informed Consent and Benefit Sharing Agreements obtained. These may be required by field-specific regulations, for example the Convention on Biological Diversity (CBD) and the associated Nagoya Protocol.

If the material used in your study was imported, please A) provide the year it was imported and B) indicate whether permits were obtained to import/export the materials used, C) provide details of any permits obtained. If this information is not available, please indicate this.

Not applicable

Not applicable

If you used archival specimens, please state how the material used in your study was acquired by the institute it is held in and provide details of any permits obtained for the original excavations/ sample collection. If this information is not available, please indicate this.

Not applicable

How was the potential cultural significance of the materials collected in your study to local communities considered in your research design? Were Indigenous peoples and/or local researchers and institutions involved with archaeological excavations / collection of specimens? If so, please provide a description of their involvement.

Not applicable

If your manuscript includes photographs of human remains please indicate whether authors obtained permission from descendants or affiliated cultural communities to do so.

Not applicable

**Questionnaire and Consent Statement**

Dear Participants,

You are warmly invited to take part in our study titled "Explaining subscription intention for video streaming platforms in China: Integrating the UTAUT2 model, perceived value theory, and S-O-R theory." This research aims to investigate the factors influencing the intention to subscribe to video streaming platforms in China. The study will also investigate the mediating effect of perceived value and the moderating effect of the Attractiveness of Alternative (AOA) on subscription intention in video streaming platforms.

Your involvement in this project will be provide details of your contact in the classification section of the survey questionnaire. Please complete all questions according to the instructions given for each section. For most questions, you will be asked to tick or check the appropriate box that best expresses your opinion. It is estimated that it will take ten minutes to complete the questionnaire, and the right to withdraw from the project at any time, including withdrawal of any information provided, and the right to withdraw from the project at any time, including withdrawal of any information provided.

In the performance of the tasks and application of the procedures there are risks of exceeding the estimated time. The results of the project may be published, but you may be assured of the complete confidentiality of data gathered in this investigation: the identity of participants will not be made public without their consent.

To ensure anonymity and confidentiality, all participants will not be recognized in the analysis as only aggregated outcome will be analyzed and presented. In our analysis and presentation of the results, we will only use aggregated data, ensuring that individual responses cannot be identified.

The research is conducted under the leadership of Dr.Chen Mobai from Qingdao University of Science and Technology (QUST), China. Should you have any questions or concerns about your participation, please do not hesitate to contact us at 18562637820@163.com or 02604@qust.edu.cn. The findings from this study may be published. This project has received approval from Qingdao University of Science and Technology Human Ethics Committee.

We appreciate your consideration and hope you will decide to contribute to this significant study.

Yours sincerely,

Best Regards

Chen Mobai & Team

Director of the Department of Film and Animation, School of Media,

Qingdao University of Science and Technology, China

**SECTION** **I:** **DEMOGRAPHIC** **PROFILE**:

1. City/district of research: China
2. Are you a existing subscriber of video streaming platforms?

□Yes(Please proceed to question 4)

□No(Please proceed to question3)

1. Do you have the intention to subscribe video streaming platforms?

□Yes(Please proceed to question 4)

□No(You can exit this survey)

1. Gender

- Female
- Male

1. Age

- 18-21
- 21-28
- 29-35
- 36 and 60
- 60 and above

1. Employment Status

- Full time employed
- Part-time employed
- Unemployed
- Retiree
- Others

1. Education Level

- High School
- College-Diploma/A-Level
- University
- Masters and above

1. Monthly allowance/Income

- Below RMB3000
- RMB3001-RMB5000
- RMB5001-RMB8000
- RMB8001-RMB10000
- Above RMB10000

**SECTION** **II:** **PERCEPTION** **OF** **RESPONDENT**

**Direction:**

**1 2 3 4 5 6 7**

|  |  |  |  |  |  |  |
| --- | --- | --- | --- | --- | --- | --- |

**Subscription Intention(SI)**

| SI1. | I will subscribe VSP (e.g. IQiYi/ Tencent/YouKu/Mango, etc) to follow up the latest drama in the future. | 1 | 2 | 3 | 4 | 5 | 6 | 7 |
| --- | --- | --- | --- | --- | --- | --- | --- | --- |
| SI2. | After having read the reviews about videos in VSP (e.g. IQiYi/ Tencent/YouKu/Mango, etc), it makes me desire to subscribe to a particular VSP. | 1 | 2 | 3 | 4 | 5 | 6 | 7 |
| SI3. | I plan to continue to use VSP (e.g. IQiYi/Tencent/YouKu/ Mango,etc) frequently to access informative and entertainment programs. | 1 | 2 | 3 | 4 | 5 | 6 | 7 |
| SI4. | I intend to continue subscribing VSP (e.g. IQiYi/ Tencent/ YouKu/ Mango, etc) in the future. | 1 | 2 | 3 | 4 | 5 | 6 | 7 |
| SI5. | I always try to use VSP (e.g. IQiYi/ Tencent/ YouKu/ Mango, etc) in my daily life. | 1 | 2 | 3 | 4 | 5 | 6 | 7 |
| SI6. | I will recommend VSP subscription to my friends or families. | 1 | 2 | 3 | 4 | 5 | 6 | 7 |
| SI7 | I will subscribe VSP to watch movies with my families. | 1 | 2 | 3 | 4 | 5 | 6 | 7 |
| SI8 | I will subscribe VSP to make it easy to stay informed about the latest movies and drama. | 1 | 2 | 3 | 4 | 5 | 6 | 7 |

**Performance Expectancy (PE)**

| PE1. | VSP makes it more convenient and useful to watch videos/films/ dramas by using video streaming transmission technology in my daily life. | 1 | 2 | 3 | 4 | 5 | 6 | 7 |
| --- | --- | --- | --- | --- | --- | --- | --- | --- |
| PE2. | Using VSP eases me to access various program channels. | 1 | 2 | 3 | 4 | 5 | 6 | 7 |
| PE3. | Using VSP increases my efficiency to watch preferred videos or films. | 1 | 2 | 3 | 4 | 5 | 6 | 7 |
| PE4. | VSP subscription is better than traditional television subscription. | 1 | 2 | 3 | 4 | 5 | 6 | 7 |
| PE5. | VSP proprietary program recommender systems make program selection relatively effortless. | 1 | 2 | 3 | 4 | 5 | 6 | 7 |
| PE6 | Using VSP improves the quality of my daily entertainment activities. | 1 | 2 | 3 | 4 | 5 | 6 | 7 |
| PE7 | VSP subscription skips advertising and improves video watching experience. | 1 | 2 | 3 | 4 | 5 | 6 | 7 |
| PE8 | VSP synopsis of videos or documentaries is useful to disseminate information to viewers. | 1 | 2 | 3 | 4 | 5 | 6 | 7 |
| PE9 | VSP bullet-screen/live comment is easier to review videos to connect with other users worldwide. | 1 | 2 | 3 | 4 | 5 | 6 | 7 |

**Effort Expectancy (EE)**

| EE1. | It is not that hard to get familiar with the basic functions of VSP, such as video playback, fast forward, bullet screen, search. | 1 | 2 | 3 | 4 | 5 | 6 | 7 |
| --- | --- | --- | --- | --- | --- | --- | --- | --- |
| EE2. | VSP is on interactive platforms, which are user-friendly and are designed to be used effortlessly by the consumers. | 1 | 2 | 3 | 4 | 5 | 6 | 7 |
| EE3. | Using VSP anywhere and anytime via multiple devices makes it easier to watch video/film/ drama. | 1 | 2 | 3 | 4 | 5 | 6 | 7 |
| EE4. | VSP enables search key words. (e.g. film/drama name or actor names, etc). | 1 | 2 | 3 | 4 | 5 | 6 | 7 |
| EE5 | VSP recommender system could automatically select videos for viewers. | 1 | 2 | 3 | 4 | 5 | 6 | 7 |
| EE6 | Interface and VSP platform function is easy to understand and operate. | 1 | 2 | 3 | 4 | 5 | 6 | 7 |
| EE7 | Multi-devices (such as telephone, laptop, computer, pad) can be used to watch video or films or dramas in VSP. | 1 | 2 | 3 | 4 | 5 | 6 | 7 |
| EE8 | VSP apps download, application and connection are easy to conduct. | 1 | 2 | 3 | 4 | 5 | 6 | 7 |
| EE9 | VSP will be highly convenient for searching for the favorite content. | 1 | 2 | 3 | 4 | 5 | 6 | 7 |

**Social Influence (SI)**

| SI1. | My relatives and friends recommend VSP to me. | 1 | 2 | 3 | 4 | 5 | 6 | 7 |
| --- | --- | --- | --- | --- | --- | --- | --- | --- |
| SI2. | Most of my friends and families have subscribed VSP. | 1 | 2 | 3 | 4 | 5 | 6 | 7 |
| SI3. | Most of my peers and friends value VSP. | 1 | 2 | 3 | 4 | 5 | 6 | 7 |
| SI4. | I use VSP because many of my friends or colleagues are using VSP. | 1 | 2 | 3 | 4 | 5 | 6 | 7 |
| SI5 | I use VSP because of recommendation of other online celebrity and WeChat groups. | 1 | 2 | 3 | 4 | 5 | 6 | 7 |
| SI6 | I’m keen to view the drama or videos that recommended by my friends and peers. | 1 | 2 | 3 | 4 | 5 | 6 | 7 |
| SI7 | Most of my friends consider VSP subscription is trendy and contemporary. | 1 | 2 | 3 | 4 | 5 | 6 | 7 |
| SI8 | Most people use VSP to watch videos in their leisure time. | 1 | 2 | 3 | 4 | 5 | 6 | 7 |

**Hedonic Motivation (HM)**

| HM1. | Watching live films and sports programs in VSP makes me feel relax and joyful. | 1 | 2 | 3 | 4 | 5 | 6 | 7 |
| --- | --- | --- | --- | --- | --- | --- | --- | --- |
| HM2. | Viewing variety shows and live broadcasting in VSP is fun. | 1 | 2 | 3 | 4 | 5 | 6 | 7 |
| HM3. | Watching cartoons and playing games or E-sports in VSP is entertaining. | 1 | 2 | 3 | 4 | 5 | 6 | 7 |
| HM4. | Communication through bullet screen to share viewing experience in VSP is enjoyable. | 1 | 2 | 3 | 4 | 5 | 6 | 7 |
| HM5 | I am satisfied with the performance of VSP. | 1 | 2 | 3 | 4 | 5 | 6 | 7 |
| HM6 | Viewing and following-up the latest drama or films in VSP can relieve the pressure of work and life. | 1 | 2 | 3 | 4 | 5 | 6 | 7 |
| HM7 | Communication through bullet screen to share viewing experience from VSP makes me delighted. | 1 | 2 | 3 | 4 | 5 | 6 | 7 |

**Habit(H)**

| H1. | The use of VSP to view and follow-up the latest drama has become a habit for me. | 1 | 2 | 3 | 4 | 5 | 6 | 7 |
| --- | --- | --- | --- | --- | --- | --- | --- | --- |
| H2. | I normally use VSP to watch films and drama at similar time of the day. | 1 | 2 | 3 | 4 | 5 | 6 | 7 |
| H3. | I get used to watch cartoon and play games via VSP. | 1 | 2 | 3 | 4 | 5 | 6 | 7 |
| H4. | Viewing various shows and live broadcasting via VSP becomes natural to me. | 1 | 2 | 3 | 4 | 5 | 6 | 7 |
| H5 | Watching films or drama via VSP has become part of my life routine that I always enjoy. | 1 | 2 | 3 | 4 | 5 | 6 | 7 |
| H6 | Using VSP to watch good dramas or films is something that I do without hesitation. | 1 | 2 | 3 | 4 | 5 | 6 | 7 |
| H7 | Communication through bullet screen to share viewing experience from VSP makes me feel delighted. | 1 | 2 | 3 | 4 | 5 | 6 | 7 |

**Perceived Value(PV)**

| PV1. | Videos in VSP have consistent quality. | 1 | 2 | 3 | 4 | 5 | 6 | 7 |
| --- | --- | --- | --- | --- | --- | --- | --- | --- |
| PV2. | Documentaries in VSP sound truthful and always reflect reality. | 1 | 2 | 3 | 4 | 5 | 6 | 7 |
| PV3. | Famous dramas in VSP always ranked well. | 1 | 2 | 3 | 4 | 5 | 6 | 7 |
| PV4. | Subscription of VSP is a good buy. | 1 | 2 | 3 | 4 | 5 | 6 | 7 |
| PV5. | VSP Subscription is reasonably priced. | 1 | 2 | 3 | 4 | 5 | 6 | 7 |
| PV6. | I think VSP provide good value for money. | 1 | 2 | 3 | 4 | 5 | 6 | 7 |
| PV7. | I feel that using VSP offers significant cost savings relative to substitute services such as Direct to Home (DTH) services. | 1 | 2 | 3 | 4 | 5 | 6 | 7 |
| PV8 | I feel socially connected and interacted while using VSP service and function. | 1 | 2 | 3 | 4 | 5 | 6 | 7 |

**Attractiveness of Alternative (AOA)**

| AOA1. | There are different VSP alternatives in the market. | 1 | 2 | 3 | 4 | 5 | 6 | 7 |
| --- | --- | --- | --- | --- | --- | --- | --- | --- |
| AOA2. | I would probably be more pleased with other VSP substitutes (e.g TikTok, Bilibili, Slogan, et al.). | 1 | 2 | 3 | 4 | 5 | 6 | 7 |
| AOA3. | There are other VSP alternatives (e.g TikTok, Bilibili, Slogan, et al.) with which I would probably be equally or more satisfied. | 1 | 2 | 3 | 4 | 5 | 6 | 7 |
| AOA4. | Using other VSP (e.g TikTok, Bilibili, Slogan, et al.) would be more satisfying than using this VSP. | 1 | 2 | 3 | 4 | 5 | 6 | 7 |
| AOA5. | Using other competing VSP (e.g TikTok, Bilibili, Slogan, et al.) would provide more benefits than using the current ones (VSP). | 1 | 2 | 3 | 4 | 5 | 6 | 7 |
| AOA6. | Other VSP alternatives (e.g TikTok, Bilibili, Slogan, et al.) may offer different packages and attractive international channels. | 1 | 2 | 3 | 4 | 5 | 6 | 7 |
| AOA7. | I know that there are VSP alternatives I can switch to. | 1 | 2 | 3 | 4 | 5 | 6 | 7 |
| AOA8. | There are other VSP alternatives that provide different service or function such as trail or different packages. | 1 | 2 | 3 | 4 | 5 | 6 | 7 |
| AOA9. | There are other VSP alternatives I find more attractive than the one I am using such as dual language subtitles and ranking. | 1 | 2 | 3 | 4 | 5 | 6 | 7 |

**Thank** **you** **for** **your** **cooperation**

Please use the space provided for any additional comments/suggestions:


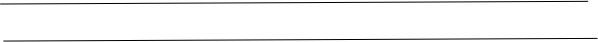


If you wish to have a specific report on the main findings of this study, please fill in the form below:

**REQUEST** **FOR** **INFORMATION**

I would like to know the result of this survey. Please send it to:

Name of respondent :

Name of company :

Mailing address :

Email :

Delivery preference :

(please tick in box)
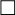
 Hard copy (post mail )
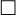
 MsWord (e-mail)

**问卷和同意书**

尊敬的参与者：

我们诚挚地邀请您参加我们的研究，题为“分析中国视频流媒体平台的订阅意愿：

整合 UTAUT2 模型、感知价值理论和 S-O-R 理论”。本研究旨在调查影响中国视频流 平台订阅意愿的因素。本研究还将调查感知价值的中介作用以及替代品吸引力（AOA）

对视频流媒体平台订阅意愿的调节作用。

您参与此项目的详细信息将在调查问卷的分类部分提供。请根据每个部分的说明完

成所有问题。对于大多数问题，您将被要求勾选或选中最能表达您意见的适当框。据估

计，完成问卷需要十分钟，有权随时退出项目，包括撤回提供的任何信息，也有权随时

撤出项目，包括收回提供的任何资料。

在执行任务和应用程序时，存在超过估计时间的风险。该项目的结果可能会公布，

但您可以放心，本次调查中收集的数据是完全保密的：未经参与者同意，参与者的身份

不会公开。

为确保匿名性和保密性，分析中不会识别所有参与者，因为只会分析和呈现汇总结

果。在我们对结果的分析和呈现中，我们将只使用汇总数据，确保无法识别单个响应。

如果您对参与有任何 疑问或疑虑，请随时通过以下方式与我们联系：18562637820@163.com 或 02604@qust.edu.cn。这项研究的结果可能会发表。该项目已获得青岛科技大学人类伦理 委员会的批准。

我们感谢您的考虑，并希望您决定为这项重要的研究做出贡献。

谢谢！

陈墨白

传媒学院电影与动画系主任

青岛科技大学, 中国

**第一部分：人口统计特征概况**

请在第一部分圈出以下每个问题的答案：

**1. 性别**

- 女性
- 男性

**2. 年龄**

- 21岁以下
- 21-28岁
- 29-35岁
- 36-45岁
- 46-59岁
- 60 岁以上

**3. 就业状况**

- 全职就业
- 兼职就业
- 未就业
- 退休
- 其他

**4. 教育水平**

- 高中及以下
- 专科
- 本科
- 研究生及以上

**5. 月收入**

- 3000人民币以下
- 3001人民币-5000人民币
- 5001人民币-8000人民币
- 8001人民币-RMB10000人民币
- 10000 人民币以上

**第二部分:**

对于每个问题，请圈出数字，表示您的同意程度。

| 1 | 2 | 3 | 4 | 5 | 6 | 7 |
| --- | --- | --- | --- | --- | --- | --- |
| 非常不同意 | 不同意 | 比较不同意 | 一般 | 比较同意 | 同意 | 非常同意 |

**订阅意向(SI)**

| S1. | 我将订阅视频流媒体平台(例如： 爱奇艺/ 腾讯/优酷/芒果, 等)来跟进未来的最新剧集。 | 1 | 2 | 3 | 4 | 5 | 6 | 7 |
| --- | --- | --- | --- | --- | --- | --- | --- | --- |
| S2. | 我在视频流媒体平台(例如： 爱奇艺/ 腾讯/优酷/芒果, 等)中阅读了有关视频的评论后，让我很想订阅某个特定的视频流媒体平台。 | 1 | 2 | 3 | 4 | 5 | 6 | 7 |
| S3. | 我计划继续使用视频流媒体平台(例如： 爱奇艺/ 腾讯/优酷/芒果, 等)来经常观看娱乐节目。 | 1 | 2 | 3 | 4 | 5 | 6 | 7 |
| S4. | 我打算在未来继续订阅视频流媒体平台(例如： 爱奇艺/ 腾讯/优酷/芒果, 等)。 | 1 | 2 | 3 | 4 | 5 | 6 | 7 |
| S5. | 我在日常生活中总是尝试使用视频流媒体平台（例如：爱奇艺/腾讯/优酷/芒果等）。 | 1 | 2 | 3 | 4 | 5 | 6 | 7 |
| S6. | 我会向我的朋友或家人推荐视频流媒体平台订阅。 | 1 | 2 | 3 | 4 | 5 | 6 | 7 |
| S7 | 我将订阅视频流媒体平台并与家人一起用视频流媒体平台看电影。 | 1 | 2 | 3 | 4 | 5 | 6 | 7 |
| S8 | 我将订阅视频流媒体平台，以便随时了解最新的电影和电视剧。 |  |  |  |  |  |  |  |

**绩效预期 (PE)**

| PE1. | 在日常生活中，视频流媒体平台使用视频流传输技术，使得用户观看视频/电影/电视剧更加方便和有用。 | 1 | 2 | 3 | 4 | 5 | 6 | 7 |
| --- | --- | --- | --- | --- | --- | --- | --- | --- |
| PE2. | 使用视频流媒体平台可以使我更方便的访问各种节目频道。 | 1 | 2 | 3 | 4 | 5 | 6 | 7 |
| PE3. | 使用视频流媒体平台可以提高观看我喜欢的视频或影片的效率。 | 1 | 2 | 3 | 4 | 5 | 6 | 7 |
| PE4. | 订阅视频流媒体平台比传统的电视订阅更好。 | 1 | 2 | 3 | 4 | 5 | 6 |  |
| PE5. | 视频流媒体平台专有的影片推荐系统使用户的影片选择相对容易。 | 1 | 2 | 3 | 4 | 5 | 6 |  |
| PE6 | 使用视频流媒体平台可以提高我的日常娱乐活动的质量。 |  |  |  |  |  |  |  |
| PE7 | 视频流媒体平台订阅可跳过广告并改善视频观看体验。 |  |  |  |  |  |  |  |
| PE8 | 视频流媒体平台的视频或纪录片预告片有助于向观众传播信息。 |  |  |  |  |  |  |  |
| PE9 | 视频流媒体平台的弹幕/实时评论功能使用户更容易观看视频，以便与其他用户建立联系。 |  |  |  |  |  |  |  |

**预期努力(EE)**

| EE1. | 熟悉视频流媒体平台的基本功能并不难，如视频播放、快进、弹幕、搜索。 | 1 | 2 | 3 | 4 | 5 | 6 | 7 |
| --- | --- | --- | --- | --- | --- | --- | --- | --- |
| EE2. | 视频流媒体平台是用户友好的交互式平台，它的设计方便消费者使用。 | 1 | 2 | 3 | 4 | 5 | 6 | 7 |
| EE3. | 通过多个设备随时随地使用视频流媒体平台，可以更轻松地观看视频/电影/电视剧。 | 1 | 2 | 3 | 4 | 5 | 6 | 7 |
| EE4. | 视频流媒体平台可以启用搜索关键字（例如电影/电视剧名称或演员名字等）。 | 1 | 2 | 3 | 4 | 5 | 6 | 7 |
| EE5 | 视频流媒体平台推荐系统可以自动为观众选择视频。 |  |  |  |  |  |  |  |
| EE6 | 视频流媒体平台的界面和功能易于理解和操作。 |  |  |  |  |  |  |  |
| EE7 | 多设备（如电话、笔记本电脑、台式电脑、平板电脑）可用于观看视频流媒体平台中的视频或电影或电视剧。 |  |  |  |  |  |  |  |
| EE8 | 视频流媒体平台的应用程序下载、申请账号和连接都很容易进行。 |  |  |  |  |  |  |  |
| EE9 | 视频流媒体平台将非常方便搜索喜爱的内容。 |  |  |  |  |  |  |  |

**社会影响 (SI)**

| SI1. | 我的亲戚朋友向我推荐视频流媒体平台。 | 1 | 2 | 3 | 4 | 5 | 6 | 7 |
| --- | --- | --- | --- | --- | --- | --- | --- | --- |
| SI2. | 我的大多数朋友和家人都订阅了视频流媒体平台。 | 1 | 2 | 3 | 4 | 5 | 6 | 7 |
| SI3. | 我的大多数同龄人和朋友都很重视视频流媒体平台。 | 1 | 2 | 3 | 4 | 5 | 6 | 7 |
| SI4. | 我之所以使用视频流媒体平台，是因为我的许多朋友或同事都在使用视频流媒体平台。 | 1 | 2 | 3 | 4 | 5 | 6 | 7 |
| SI5 | 我之所以使用视频流媒体平台，是因为其他网红和微信群的推荐。 |  |  |  |  |  |  |  |
| SI6 | 我很喜欢看朋友和同龄人推荐的电视剧、电影或视频。 |  |  |  |  |  |  |  |
| SI7 | 我的大多数朋友都认为订阅视频流媒体平台是时尚和现代的。 |  |  |  |  |  |  |  |
| SI8 | 大多数人在业余时间使用视频流媒体平台观看视频。 |  |  |  |  |  |  |  |

**享乐动机 (HM)**

| HM1. | 在视频流媒体平台中观看现场电影和体育节目让我感到放松和快乐。 | 1 | 2 | 3 | 4 | 5 | 6 | 7 |
| --- | --- | --- | --- | --- | --- | --- | --- | --- |
| HM2. | 在视频流媒体平台中观看综艺节目和现场直播很有趣。 | 1 | 2 | 3 | 4 | 5 | 6 | 7 |
| HM3. | 在视频流媒体平台中看动画片、玩游戏或电子竞技很有趣。 | 1 | 2 | 3 | 4 | 5 | 6 | 7 |
| HM4. | 通过弹幕交流，分享视频流媒体平台中的观看体验，令人愉快。 | 1 | 2 | 3 | 4 | 5 | 6 | 7 |
| HM5 | 我对视频流媒体平台的性能感到满意。 |  |  |  |  |  |  |  |
| HM6 | 在视频流媒体平台观看和跟进最新的电视剧或电影可以缓解工作和生活的压力。 |  |  |  |  |  |  |  |
| HM7 | 通过弹幕交流，分享视频流媒体平台的观看体验，让我很高兴。 |  |  |  |  |  |  |  |

**习惯(H)**

| H1. | 使用视频流媒体平台观看和追踪最新的电视剧、电影已经成为我的一种习惯。 | 1 | 2 | 3 | 4 | 5 | 6 | 7 |
| --- | --- | --- | --- | --- | --- | --- | --- | --- |
| H2. | 我通常在一天中的相同时间使用用视频流媒体平台观看电影和电视剧。 | 1 | 2 | 3 | 4 | 5 | 6 | 7 |
| H3. | 我习惯了通过视频流媒体平台观看动画片和玩游戏。 | 1 | 2 | 3 | 4 | 5 | 6 | 7 |
| H4. | 通过视频流媒体平台来观看各种节目和直播对我来说很平常。 | 1 | 2 | 3 | 4 | 5 | 6 | 7 |
| H5 | 通过视频流媒体平台来观看电影或电视剧已经成为我日常生活的一部分，我一直很喜欢。 |  |  |  |  |  |  |  |
| H6 | 我毫不犹豫地选择使用视频流媒体平台来观看电视剧或电影。 |  |  |  |  |  |  |  |
| H7 | 我很高兴通过视频流媒体平台的弹幕交流，并分享观看体验。 |  |  |  |  |  |  |  |

**感知价值(PV)**

| PV1. | 视频流媒体平台中的视频具有一致的质量。 | 1 | 2 | 3 | 4 | 5 | 6 | 7 |
| --- | --- | --- | --- | --- | --- | --- | --- | --- |
| PV2. | 视频流媒体平台中的纪录片看起来很真实，并且它总是反映现实。 | 1 | 2 | 3 | 4 | 5 | 6 | 7 |
| PV3. | 视频流媒体平台中的著名的电影或电视剧总是名列前茅。 | 1 | 2 | 3 | 4 | 5 | 6 | 7 |
| PV4. | 订阅视频流媒体平台的价格很划算。 |  |  |  |  |  |  |  |
| PV5. | 订阅视频流媒体平台价格合理。 |  |  |  |  |  |  |  |
| PV6. | 我认为视频流媒体平台性价比很高。 |  |  |  |  |  |  |  |
| PV7. | 我觉得与卫星直播电视（DTH）服务等替代服务相比，使用视频流媒体平台可以显著节省成本。 |  |  |  |  |  |  |  |
| PV8 | 在使用视频流媒体平台的服务和功能时，我感觉到了社交联系和互动。 |  |  |  |  |  |  |  |

**备选方案的吸引力 (AOA)**

| AOA1. | 市场上有不同的可供选择的多种视频流媒体平台。 | 1 | 2 | 3 | 4 | 5 | 6 | 7 |
| --- | --- | --- | --- | --- | --- | --- | --- | --- |
| AOA2. | 我可能会更高兴选择其他视频流媒体平台（例如TikTok、哔哩哔哩、西瓜视频等）。 | 1 | 2 | 3 | 4 | 5 | 6 | 7 |
| AOA3. | 我可能会对其他视频流媒体平台同样满意或更满意（例如TikTok、哔哩哔哩、西瓜视频等）。 | 1 | 2 | 3 | 4 | 5 | 6 | 7 |
| AOA4. | 使用其他视频流媒体平台（例如TikTok、哔哩哔哩、西瓜视频等）将比使用此视频流媒体平台更令人满意。 | 1 | 2 | 3 | 4 | 5 | 6 | 7 |
| AOA5. | 使用其他竞争性视频流媒体平台（如TikTok、哔哩哔哩、西瓜视频等）将比使用现有视频流媒体平台带来更多好处。 |  |  |  |  |  |  |  |
| AOA6. | 其他视频流媒体平台替代方案（如TikTok、哔哩哔哩、西瓜视频等）可能提供不同的套餐和有吸引力的国际频道。 |  |  |  |  |  |  |  |
| AOA7. | 我知道我可以切换到视频流媒体平台的替代方案。 |  |  |  |  |  |  |  |
| AOA8. | 还有其他不同的视频流媒体平台提供不同的服务或功能，例如预告片或套餐。 |  |  |  |  |  |  |  |
| AOA9. | 我发现还有其他视频流媒体平台替代方案比我现在使用的视频流媒体平台更具吸引力，比如双语字幕和排名。 |  |  |  |  |  |  |  |

**Note: VSP=Video streaming platform**

**标识变量**

**社会国家形象**

| H1. | 你对马尔代夫的印象是它一个保障个人权利和自由的国家 | 1 | 2 | 3 | 4 | 5 | 6 | 7 |
| --- | --- | --- | --- | --- | --- | --- | --- | --- |
| H2. | 你对马尔代夫的印象是它是一个尊重“职业道德”（公平）原则的国家。 | 1 | 2 | 3 | 4 | 5 | 6 | 7 |
| H3. | 你对马尔代夫的印象是它是一个促进公益事业（即对社会负责）的国家。 | 1 | 2 | 3 | 4 | 5 | 6 | 7 |
| H4. | 你对马尔代夫的印象是它是一个负责任的国际社会成员。 | 1 | 2 | 3 | 4 | 5 | 6 | 7 |

**谢谢您的合作**

请在提供的空白处添加任何其他意见/建议：如果您希望获得关于本研究主要发现的具体报告，请填写下表：

**信息请求**

我想知道这项调查的结果。请发送至：

被申请人姓名：

单位名称：

邮寄地址：

电子邮件：
