# Supplementary material for: Explaining subscription intention for video streaming platforms in China: Integrating the UTAUT2 model, perceived value theory, and S-O-R theory
Source: PLoS One. 2025 May 8;20(5):e0322860. doi: 10.1371/journal.pone.0322860 (PMC12061175; doi:10.1371/journal.pone.0322860)
Supplement: S2 — (DOCX) [file pone.0322860.s002.docx]

**Questionnaire**

**SECTION** **I:** **DEMOGRAPHIC** **PROFILE**:

1. City/district of research: China
2. Are you a existing subscriber of video streaming platforms?

□Yes(Please proceed to question 4)

□No(Please proceed to question3)

1. Do you have the intention to subscribe video streaming platforms?

□Yes(Please proceed to question 4)

□No(You can exit this survey)

1. Gender

- Female
- Male

1. Age

- 18-21
- 21-28
- 29-35
- 36 and 60
- 60 and above

1. Employment Status

- Full time employed
- Part-time employed
- Unemployed
- Retiree
- Others

1. Education Level

- High School
- College-Diploma/A-Level
- University
- Masters and above

1. Monthly allowance/Income

- Below RMB3000
- RMB3001-RMB5000
- RMB5001-RMB8000
- RMB8001-RMB10000
- Above RMB10000

**SECTION** **II:** **PERCEPTION** **OF** **RESPONDENT**

**Direction:**

**1 2 3 4 5 6 7**

|  |  |  |  |  |  |  |
| --- | --- | --- | --- | --- | --- | --- |

**Subscription Intention(SI)**

| SI1. | I will subscribe VSP (e.g. IQiYi/ Tencent/YouKu/Mango, etc) to follow up the latest drama in the future. | 1 | 2 | 3 | 4 | 5 | 6 | 7 |
| --- | --- | --- | --- | --- | --- | --- | --- | --- |
| SI2. | After having read the reviews about videos in VSP (e.g. IQiYi/ Tencent/YouKu/Mango, etc), it makes me desire to subscribe to a particular VSP. | 1 | 2 | 3 | 4 | 5 | 6 | 7 |
| SI3. | I plan to continue to use VSP (e.g. IQiYi/Tencent/YouKu/ Mango,etc) frequently to access informative and entertainment programs. | 1 | 2 | 3 | 4 | 5 | 6 | 7 |
| SI4. | I intend to continue subscribing VSP (e.g. IQiYi/ Tencent/ YouKu/ Mango, etc) in the future. | 1 | 2 | 3 | 4 | 5 | 6 | 7 |
| SI5. | I always try to use VSP (e.g. IQiYi/ Tencent/ YouKu/ Mango, etc) in my daily life. | 1 | 2 | 3 | 4 | 5 | 6 | 7 |
| SI6. | I will recommend VSP subscription to my friends or families. | 1 | 2 | 3 | 4 | 5 | 6 | 7 |
| SI7 | I will subscribe VSP to watch movies with my families. | 1 | 2 | 3 | 4 | 5 | 6 | 7 |
| SI8 | I will subscribe VSP to make it easy to stay informed about the latest movies and drama. | 1 | 2 | 3 | 4 | 5 | 6 | 7 |

Venkatesh et al. (2012)

**Performance Expectancy (PE)**

| PE1. | VSP makes it more convenient and useful to watch videos/films/ dramas by using video streaming transmission technology in my daily life. | 1 | 2 | 3 | 4 | 5 | 6 | 7 |
| --- | --- | --- | --- | --- | --- | --- | --- | --- |
| PE2. | Using VSP eases me to access various program channels. | 1 | 2 | 3 | 4 | 5 | 6 | 7 |
| PE3. | Using VSP increases my efficiency to watch preferred videos or films. | 1 | 2 | 3 | 4 | 5 | 6 | 7 |
| PE4. | VSP subscription is better than traditional television subscription. | 1 | 2 | 3 | 4 | 5 | 6 | 7 |
| PE5. | VSP proprietary program recommender systems make program selection relatively effortless. | 1 | 2 | 3 | 4 | 5 | 6 | 7 |
| PE6 | Using VSP improves the quality of my daily entertainment activities. | 1 | 2 | 3 | 4 | 5 | 6 | 7 |
| PE7 | VSP subscription skips advertising and improves video watching experience. | 1 | 2 | 3 | 4 | 5 | 6 | 7 |
| PE8 | VSP synopsis of videos or documentaries is useful to disseminate information to viewers. | 1 | 2 | 3 | 4 | 5 | 6 | 7 |
| PE9 | VSP bullet-screen/live comment is easier to review videos to connect with other users worldwide. | 1 | 2 | 3 | 4 | 5 | 6 | 7 |

Venkatesh et al. (2012)

**Effort Expectancy (EE)**

| EE1. | It is not that hard to get familiar with the basic functions of VSP, such as video playback, fast forward, bullet screen, search. | 1 | 2 | 3 | 4 | 5 | 6 | 7 |
| --- | --- | --- | --- | --- | --- | --- | --- | --- |
| EE2. | VSP is on interactive platforms, which are user-friendly and are designed to be used effortlessly by the consumers. | 1 | 2 | 3 | 4 | 5 | 6 | 7 |
| EE3. | Using VSP anywhere and anytime via multiple devices makes it easier to watch video/film/ drama. | 1 | 2 | 3 | 4 | 5 | 6 | 7 |
| EE4. | VSP enables search key words. (e.g. film/drama name or actor names, etc). | 1 | 2 | 3 | 4 | 5 | 6 | 7 |
| EE5 | VSP recommender system could automatically select videos for viewers. | 1 | 2 | 3 | 4 | 5 | 6 | 7 |
| EE6 | Interface and VSP platform function is easy to understand and operate. | 1 | 2 | 3 | 4 | 5 | 6 | 7 |
| EE7 | Multi-devices (such as telephone, laptop, computer, pad) can be used to watch video or films or dramas in VSP. | 1 | 2 | 3 | 4 | 5 | 6 | 7 |
| EE8 | VSP apps download, application and connection are easy to conduct. | 1 | 2 | 3 | 4 | 5 | 6 | 7 |
| EE9 | VSP will be highly convenient for searching for the favorite content. | 1 | 2 | 3 | 4 | 5 | 6 | 7 |

Venkatesh et al. (2012)

**Social Influence (SI)**

| SI1. | My relatives and friends recommend VSP to me. | 1 | 2 | 3 | 4 | 5 | 6 | 7 |
| --- | --- | --- | --- | --- | --- | --- | --- | --- |
| SI2. | Most of my friends and families have subscribed VSP. | 1 | 2 | 3 | 4 | 5 | 6 | 7 |
| SI3. | Most of my peers and friends value VSP. | 1 | 2 | 3 | 4 | 5 | 6 | 7 |
| SI4. | I use VSP because many of my friends or colleagues are using VSP. | 1 | 2 | 3 | 4 | 5 | 6 | 7 |
| SI5 | I use VSP because of recommendation of other online celebrity and WeChat groups. | 1 | 2 | 3 | 4 | 5 | 6 | 7 |
| SI6 | I’m keen to view the drama or videos that recommended by my friends and peers. | 1 | 2 | 3 | 4 | 5 | 6 | 7 |
| SI7 | Most of my friends consider VSP subscription is trendy and contemporary. | 1 | 2 | 3 | 4 | 5 | 6 | 7 |
| SI8 | Most people use VSP to watch videos in their leisure time. | 1 | 2 | 3 | 4 | 5 | 6 | 7 |

Venkatesh et al. (2012)

**Hedonic Motivation (HM)**

| HM1. | Watching live films and sports programs in VSP makes me feel relax and joyful. | 1 | 2 | 3 | 4 | 5 | 6 | 7 |
| --- | --- | --- | --- | --- | --- | --- | --- | --- |
| HM2. | Viewing variety shows and live broadcasting in VSP is fun. | 1 | 2 | 3 | 4 | 5 | 6 | 7 |
| HM3. | Watching cartoons and playing games or E-sports in VSP is entertaining. | 1 | 2 | 3 | 4 | 5 | 6 | 7 |
| HM4. | Communication through bullet screen to share viewing experience in VSP is enjoyable. | 1 | 2 | 3 | 4 | 5 | 6 | 7 |
| HM5 | I am satisfied with the performance of VSP. | 1 | 2 | 3 | 4 | 5 | 6 | 7 |
| HM6 | Viewing and following-up the latest drama or films in VSP can relieve the pressure of work and life. | 1 | 2 | 3 | 4 | 5 | 6 | 7 |
| HM7 | Communication through bullet screen to share viewing experience from VSP makes me delighted. | 1 | 2 | 3 | 4 | 5 | 6 | 7 |

Kim et al. (2006);Venkatesh et al. (2012)

**Habit(H)**

| H1. | The use of VSP to view and follow-up the latest drama has become a habit for me. | 1 | 2 | 3 | 4 | 5 | 6 | 7 |
| --- | --- | --- | --- | --- | --- | --- | --- | --- |
| H2. | I normally use VSP to watch films and drama at similar time of the day. | 1 | 2 | 3 | 4 | 5 | 6 | 7 |
| H3. | I get used to watch cartoon and play games via VSP. | 1 | 2 | 3 | 4 | 5 | 6 | 7 |
| H4. | Viewing various shows and live broadcasting via VSP becomes natural to me. | 1 | 2 | 3 | 4 | 5 | 6 | 7 |
| H5 | Watching films or drama via VSP has become part of my life routine that I always enjoy. | 1 | 2 | 3 | 4 | 5 | 6 | 7 |
| H6 | Using VSP to watch good dramas or films is something that I do without hesitation. | 1 | 2 | 3 | 4 | 5 | 6 | 7 |
| H7 | Communication through bullet screen to share viewing experience from VSP makes me feel delighted. | 1 | 2 | 3 | 4 | 5 | 6 | 7 |

Limayem, Hirt & Cheung.(2007) ; Venkatesh et al. (2012)

**Perceived Value(PV)**

| PV1. | Videos in VSP have consistent quality. | 1 | 2 | 3 | 4 | 5 | 6 | 7 |
| --- | --- | --- | --- | --- | --- | --- | --- | --- |
| PV2. | Documentaries in VSP sound truthful and always reflect reality. | 1 | 2 | 3 | 4 | 5 | 6 | 7 |
| PV3. | Famous dramas in VSP always ranked well. | 1 | 2 | 3 | 4 | 5 | 6 | 7 |
| PV4. | Subscription of VSP is a good buy. | 1 | 2 | 3 | 4 | 5 | 6 | 7 |
| PV5. | VSP Subscription is reasonably priced. | 1 | 2 | 3 | 4 | 5 | 6 | 7 |
| PV6. | I think VSP provide good value for money. | 1 | 2 | 3 | 4 | 5 | 6 | 7 |
| PV7. | I feel that using VSP offers significant cost savings relative to substitute services such as Direct to Home (DTH) services. | 1 | 2 | 3 | 4 | 5 | 6 | 7 |
| PV8 | I feel socially connected and interacted while using VSP service and function. | 1 | 2 | 3 | 4 | 5 | 6 | 7 |

Zhang et al. (2024); Sweeney and Soutar (2001); Lee and Overby (2004)

**Attractiveness of Alternative (AOA)**

| AOA1. | There are different VSP alternatives in the market. | 1 | 2 | 3 | 4 | 5 | 6 | 7 |
| --- | --- | --- | --- | --- | --- | --- | --- | --- |
| AOA2. | I would probably be more pleased with other VSP substitutes (e.g TikTok, Bilibili, Slogan, et al.). | 1 | 2 | 3 | 4 | 5 | 6 | 7 |
| AOA3. | There are other VSP alternatives (e.g TikTok, Bilibili, Slogan, et al.) with which I would probably be equally or more satisfied. | 1 | 2 | 3 | 4 | 5 | 6 | 7 |
| AOA4. | Using other VSP (e.g TikTok, Bilibili, Slogan, et al.) would be more satisfying than using this VSP. | 1 | 2 | 3 | 4 | 5 | 6 | 7 |
| AOA5. | Using other competing VSP (e.g TikTok, Bilibili, Slogan, et al.) would provide more benefits than using the current ones (VSP). | 1 | 2 | 3 | 4 | 5 | 6 | 7 |
| AOA6. | Other VSP alternatives (e.g TikTok, Bilibili, Slogan, et al.) may offer different packages and attractive international channels. | 1 | 2 | 3 | 4 | 5 | 6 | 7 |
| AOA7. | I know that there are VSP alternatives I can switch to. | 1 | 2 | 3 | 4 | 5 | 6 | 7 |
| AOA8. | There are other VSP alternatives that provide different service or function such as trail or different packages. | 1 | 2 | 3 | 4 | 5 | 6 | 7 |
| AOA9. | There are other VSP alternatives I find more attractive than the one I am using such as dual language subtitles and ranking. | 1 | 2 | 3 | 4 | 5 | 6 | 7 |

**Note: VSP=Video streaming platform**

Kuo et al. (2013), Jones et al. (2000), and Kim et al. (2011)

**Thank** **you** **for** **your** **cooperation**

Please use the space provided for any additional comments/suggestions:


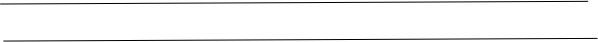


If you wish to have a specific report on the main findings of this study, please fill in the form below:

**REQUEST** **FOR** **INFORMATION**

I would like to know the result of this survey. Please send it to:

Name of respondent :

Name of company :

Mailing address :

Email :

Delivery preference :

(please tick in box)
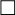
 Hard copy (post mail )
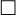
 MsWord (e-mail)
