# Supplementary material for: Explaining subscription intention for video streaming platforms in China: Integrating the UTAUT2 model, perceived value theory, and S-O-R theory
Source: PLoS One. 2025 May 8;20(5):e0322860. doi: 10.1371/journal.pone.0322860 (PMC12061175; doi:10.1371/journal.pone.0322860)
Supplement: Attachment — Submitted filename: Response to Reviewers.docx [file pone.0322860.s004.docx]

**Rebuttal Letter**

Dear Editor and Reviewers,

We would like to express our sincere gratitude for your valuable comments and constructive suggestions. Your feedback is very helpful in guiding us through the manuscript amendments. We have resubmitted the updated manuscript for your kind review. Thank you very much for offering us the opportunity to improve our work. Please refer to the marked contents and sections in the 'Revised Manuscript with Track Changes'. Author action of each comment is presented in the following sections:

Thank you

Kind regards

Author Team

**Editor’s comments**

1. Please ensure that your manuscript meets PLOS ONE's style requirements, including those for file naming.

Author Action: Thanks for your valuable comments. I rechecked the PLOS ONE style requirements and file name, revised title accordingly. I revised the heading size and format, figure files formats (Resubmitted figure file), reference citations format according to MANUSCRIPT BODY FORMATTING GUIDELINES. And I added author information in the first page of manuscript line 1-13. In addition, I revised the format of file of Title_Author_Affiliations and resubmitted again.

2. Please include a complete copy of PLOS’ questionnaire on inclusivity in global research in your revised manuscript.

Author action: Thanks for your valuable comments. I added this study added details as to who granted permissions and/or consent for the study to take place in the section of inclusivity in global research, part of Methodology, line 8-11, which is indicated as “Additional information regarding the ethical, cultural, and scientific considerations specific to inclusivity in global research is included in the Supporting Information (S4_Appendix 1 Checklist). This study was granted approval by the Human Ethics Committee (HEC), School of Communication, Qingdao University of Science and Technology, China.” The HEC approval letter, informed consent form, survey cover page, and the full questionnaire (in both Mandarin and English) are included in S4_Appendix 1: Questionnaire as supporting information.

3. Please ensure that you include a title page within your main document.

Author Action: Thanks for your valuable comments. I added title page in first page of manuscript, line 1-13.

4. As an additional recommendation to facilitate readability, Table 6 could also include the level of acceptance/ rejection of each hypothesis.

Author Action: Thanks for your valuable comments. I added results which indicated supported or not supported in section of 4.0 Results, line 1, table 6.

**Reviewer 1 Comments**:

1. Theoretical Integration and Clarification: The paper proposes an integration of UTAUT2, perceived value theory, and S-O-R theory. However, the linkage between these theories is not sufficiently clarified. How do these theories synergistically enhance our understanding of subscription intention? Provide a more detailed rationale for this theoretical integration.

# Author Action: Thanks for your valuable commets. To address the comments, we revised the entire manuscript. I added the detailed rationale for theoretical integration in the theoretical background section, behind 2.3 Moderating role of attractiveness of alternatives, pharagraph 6, line 4-13, stated as “Currently, regarding new media, limited research exists about subscription intention for SVOD, particularly for video streaming platforms in China. There is a lack of academic research on viewers’ subscription behavior of video streaming platforms in China. However, it is noted that there is a gap in the current literature in terms of understanding the drivers and determinants of viewers’ use of SVOD services [7]. Thus, this research examines the mechanism through factors influence users’ subscription decision. However, none of three theories (UTAUT2, perceived value theory, S-O-R model) can comprehensively explain the circumstances in this research. Based on the identified gap in the literature, this study concludes that a single theory is not sufficient to address the current research problem. Thus, this study addresses the gap in existing literature regarding viewers’ decision-making process when subscribing to video streaming platforms in China by integration of UTAUT2, perceived value theory, and S-O-R theory.” And added contents in theoretical background section, behind 2.3 Moderating role of attractiveness of alternatives, pharagraph 7, line 14-26, stated as “In the context of video streaming platforms, the UTAUT2 model highlights the roles of factors such as performance expectancy, effort expectancy, social influence, hedonic motivation, and habit in shaping users' subscription intentions [84, 90]. Futhermore, this study fills a research gap in the literature, especially regarding the mediating role of perceived value in the relationships between performance expectancy, effort expectancy, social influence, hedonic motivation, and subscription intention in video streaming platforms by the basis of the Perceived Value Theory [79] and S-O-R (Stimulus-Organism-Response) model [19, 54, 91]. First, this study extends the research based on multi-dimensional perceived value in the context of video streaming platforms by Perceived Value Theory. Secondly, this research expands the S-O-R model to apply the technology acceptance variables to stimulus, which is performance expectancy, effort expectancy, social influence and hedonic motivation for viewers and expands the organism of S-O-R to perceived value. Besides, this research expands the response of S-O-R to subscription intention. Moreover, this research examines the moderating effect of the attractiveness of alternatives on the relationship between perceived value and subscription intention [36, 57]. Figure 1 is the research framework.”.

2. Operationalization of Constructs: The operational definitions of some constructs, such as perceived value and attractiveness of alternatives, could benefit from further elaboration. How do these constructs operationalize within the context of Chinese video streaming platforms? Clarify and justify the dimensions chosen.

## Author Action: Thanks for your valuable commets. To address the comments, we revised the entire manuscript. I added the contents of definition of perceived value and the constructs operationalization by multiple dimentions, which is added in theoretical background section, 2.2 Mediating role of perceived value, pharagraph 2, line 13-24, stated as “The concept of consumer perceived value in video streaming platforms pertains to the degree to which users assess the benefits and costs of such platforms about one another. The degree of success and acceptance of a technology or service is contingent upon the value consumers perceive or anticipate from streaming service [107]. The multi-dimensional aspects of perceived value are used in this research: emotional value, quality value, monetary value, and convenience value. In this study, emotional value is crucial for video streaming services as it provides a fun or enjoyable service experience to the viewers. In addition, monetary value measures or compares the cost of streaming services with traditional media devices. The quality value measures the video content and video quality which have become increasingly important for viewers to subscribe to video streaming platforms. Furthermore, the convenience value of video streaming services make it convenient, fast, and easy to watch videos on multiple devices, anytime and anywhere. Moreover, the social value of services which through the bullet screen, viewers of video streaming platforms can watch movies and talk to each other at any time [19, 59, 79].”Furthermore, I added the contents of definition of attractiveness of alternatives and the constructs operationalization by multiple dimentions, which is added in theoretical background section, 2.3 Moderation role of attractiveness of alternatives, pharagraph 2, line 20-21, stated as “The extent to which customers perceive viable competitive alternatives in the market is the attractiveness of alternatives.” And theoretical background section, 2.3 Moderation role of attractiveness of alternatives, pharagraph 4, line 27-32, stated as “In this study, the moderation effect of attractiveness of alternatives is important in influencing the subscription intention of video streaming platforms. There are several dimensionals of attractiveness of alternative in this study. VSP alternatives (e.g TikTok, Bilibili, Slogan, et al.) may offer different packages and attractive international channels. In addition, VSP alternatives that provide different service or function such as trail or different packages. Furthermore, VSP alternatives are more attractive for dual language subtitles and ranking [69, 88].”

3. Sampling Methodology: The paper uses a combination of self-selection, volunteer, and convenience sampling methods, which could introduce bias. Discuss the potential implications of this sampling strategy on the generalizability of the findings and consider stratified random sampling for future research to enhance representativeness.

Author Action: Thanks for your valuable comments. To address the comments, we revised the entire manuscript. I added potential implications of this sampling strategy on the generalizability of the findings and consider stratified random sampling for future research to enhance representativeness in section of Limitations and future research, pharagraph 3, line 28-31, stated as “Furthermore, in sampling methodology, self-selection, volunteer, and convenience sampling methods are non-probability sampling, which include an element of subjective judgment and cannot infer the overall population quantitatively. Thus, in future research, it can choose to use stratified random sampling to improve the representativeness of the research.”

And, volunteer sampling technique was chosen by the researcher of this current study, because some filtering questions in Appendix 2, were applied to select the most appropriate samples from the target population, to meet the researcher’s interests regarding the objectives of this study. In convenience sampling, the advantages are convenient, simple, and easy to implement, saving time and money. In addition, convenience sampling can be used when studying issues such as psychological mechanisms and processes where individual differences are small, the total population is unknown, and random sampling is difficult. Furthermore, this study use marker variable to minimize the potential generalization problems in this study.

4. Measurement Scales: You have adapted items from different sources for your constructs. Please ensure that these items are culturally appropriate for the Chinese market and that any necessary translations are validated.

Author Action: Thanks for your valuable comments. I have been rechecked all items of measurement scales to be suitable for Chinese market and rechecked the translations, which is validated. And I added the questionnaire within my manuscript in appendix 1.

5. Data Analysis: While PLS-SEM is used, there is an opportunity to deepen the analysis. Consider performing multi-group analysis to explore potential differences in subscription intention across different demographic groups (e.g., age, income level).

Author Action: Thanks for your variable comments. According to your great advices, this study performed multi-group analysis to explore potential differences in subscription intention across income level, gender and age of different demographic groups. However, I found out the outcome of MGA is insignificant.

For age groups of 18-45 and age above 45, the result of MGA is insignificant.

For gender groups of female and male to run MGA, the result indicated the multi-group analysis is insignificant.

For different income level of demographic groups, the result of MGA is insignificant.

6. Mediation Analysis: The mediation effect of perceived value is indicated, but the paper could benefit from a more nuanced exploration of the indirect effects. Specifically, consider the possibility of partial or full mediation and explore this in greater depth.

Author action: Thanks for your variable comments. To address the comments, I deleted the contents in Results section, the part of mediation analysis, phragraph 2, line 27-31, which indicated as “~~Considering the significant relationship between performance expectancy (β = 0.106, t = 3.277, p < 0.01), effort expectancy (β = 0.110, t = 3.001, p < 0.01), social influence (β = 0.094, t = 2.612, p < 0.01), and hedonic motivation (β = 0.134, t = 3.845, p < 0.001) with subscription intention in the moderated mediation model, perceived value partially mediate these relationships.”.~~ And I further added the contents of detailed explaination of indirect effects and consider the possiblity of partial or full mediation to further investigate it, which in Results section, the part of mediation analysis, phragraph 2, line 31-42, which indicated as “First, mediation analysis was performed to assess the mediating role of PV in the relationship between PE and S, EE and S, SI and S, HM and S. The results (see Table 6) revealed a significant indirect effect of PE on S through PV (H6: β = 0.026, t = 2.355, p < 0.01), EE on S through PV (H7: β = 0.058, t = 4.380, p < 0.001), SI on S through PV (H8, β = 0.041, t = 3.388, p < 0.001), HM on S through PV (H9, β = 0.033, t = 2.773, p < 0.01). The total effect of PE on S was significant (β = 0.132, t = 3.878, p < 0.001), with inclusion of the mediator the effect of PE on S was still significant (β = 0.106, t = 3.277, p < 0.01). The total effect of EE on S was significant (β = 0.168, t = 4.493, p < 0.001), with inclusion of the mediator the effect of EE on S was still significant (β = 0.110, t = 3.001, p < 0.01). The total effect of SI on S was significant (β = 0.135, t = 3.651, p < 0.001), with inclusion of the mediator the effect of SI on S was still significant (β = 0.094, t = 2.612, p < 0.01). The total effect of HM on S was significant (β = 0.167, t = 4.736, p < 0.001), with inclusion of the mediator the effect of HM on S was still significant (β = 0.134, t = 3.845, p < 0.001). This shows a complementary partial mediation role of perceived value in the relationship between PE and S, EE and S, SI and S, HM and S. Hense, H6, H7, H8 and H9 was supported.”

7. Moderation Analysis: The moderating effect of attractiveness of alternatives is a key contribution of your paper. Provide a more in-depth discussion of the implications of this finding, particularly how service providers might address the challenge of alternative attractiveness.

Author action: Thanks for your valuable comments. I added more in depth discussion of the implications of this finding in the part of Implications, pharagraph 5, line 20-25, which is indicated as “In addition, the results of this research may help video streaming platforms to improve viewers’perceived value, to create interface functions by multiple devices, and to improve the functions of the VSP recommender system [74]. The findings of this research can aid video streaming platforms in enhancing viewers' perceived value of VSPs through influencers such as friends or family members, other online celebrities, and social media. This, in turn, can drive an increase in subscribers to video streaming platforms.”

8. Results Presentation: The results are presented concisely, but the paper could benefit from additional visual aids such as graphs to illustrate key relationships and interactions more clearly.

Author action: Thanks for your valuable comments. To address the comments, I added the Figure 3: Research model with path coefficients (outer loadings) and R² values with moderating variable interaction terms in section of Results.

9. Implications for Practice: The implications for video streaming platforms are mentioned, but they could be more actionable. Provide specific strategies that platforms can use to enhance perceived value and mitigate the impact of attractive alternatives.

Author action: Thanks for your valuable comments. I added strategies that platforms can use to enhance perceived value to increase subscription intention in section of implications, pharagraph 5, line 13-20, which is indicated as “Secondly, the significant implication for video streaming platforms is to become viewer-oriented to better understand viewers’ preference and desires. The results of this research suggest that VSPs create consistent quality videos, dramas, or films in order to increase consumer perceived value and, thereby, subscription intention. The findings of this research can assist video streaming platforms in enhancing viewers’ perceived value of VSPs [95]. This can be achieved through the implementation of new streaming media technologies, enhanced subscription services allowing ad skipping to improve the video-watching experience, and the incorporation of features such as bullet-screen/ live comments. These improvements can attract a larger audience to subscribe to VSPs.

And added contents in section of implications, pharagraph 6, line 26-31, which is indicated as “The third significant implication for VSPs is to understand better their viewers’ practical concerns and retain or improve their subscription rate. The results of this research may help video streaming platforms understand that improving perceived value for viewers is the key factor to increase subscription intention. In addition, video streaming platforms can improve the quality and variety of content in VSPs to stimulate viewers’ hedonic motivation and thereby increase their satisfaction and gratification. Then, more viewers will choose to subscribe [105].”

In addition, I added the strategies that platforms can use to mitigate the impact of attractive alternatives in section of implications, pharagraph 7, line 34-39, which is indicated as “The fourth significant implication for the platforms and industry regards the the negative moderating effect that the attraction of alternatives (AOA) has on subscription intention. If there are too many VSP competitors, then subscription intention will be decreased. Thus, competition should occur between only a few monopoly VSPs rather than many small VSPs [88]. In summary, a smaller number of competing VSPs would increase overall subscription intention.” And added contents in section of implications, pharagraph 7, line 41, 1-5, “In addition, to mitigate the effect of alternate attractiveness of video streaming platform competitors on subscription intention. Video streaming platforms must improve their essential capabilities. Additionally, they utilize user profiles for distinguishing their key user groups [104]. Furthermore, video streaming platforms must give a positive user experience. And, they must have a comprehensive understanding of users' demands and conduct in-depth analyses of its target users' interests, desires, and habits in order to develop content that satisfies their requirements [78].”

10. Limitations and Future Research: The limitations are acknowledged; however, expand on how these limitations could specifically affect the findings of the study. Furthermore, propose clear directions for future research, including potential longitudinal studies to assess changes in subscription intention over time.

Author action: Thanks for your valuable comments. I added contents which specifically affect the findings of the study and propose clear directions for future research in section of 7. Limitations and future research, pharagraph 2, line 13-15 and line 18-19, which indicated as “Additionally, examining the same hypotheses in different countries may yield different results in terms of different consumer subscription behaviors due to cultural, ethnic, and demographic differences.” and “Furthermore, in future study, it should be added potential longitudinal studies to assess changes in subscription intention over time.” And added contents in section of Limitations and future research, pharagraph 3, line 22-24, which indicated as “However, in consumer behavior research, quantitative studies may fail to capture the deeper motivations and specific social contexts of consumer behavior, resulting in findings that lack depth and utility.” And added contents in section of Limitations and future research, pharagraph 4, line 32-34 and line 38, which indicated as “In addition, relying only on the sample of user groups and the influences of the research framework may lead to biased results, and the influences of video streaming platforms and industry practitioners may also affect the results of the study. ” and “create a new research framework model ”.

11. Ethical Considerations: It is good that ethical considerations have been addressed, but ensure that informed consent procedures are robust and that participant anonymity is maintained throughout the research process.

Author action: Thanks for your valuable comments. I added these contents in the part of Methodology, Ethical consideration, line 1-2, which indicated as “Furthermore, in this study the inform consent procedures are robust and participant anonymity is maintained throughout the research process.”.

12. References and Citations: Ensure that all references are current and relevant, and that citations are consistently formatted according to the appropriate academic style guide.

Author action: Thanks for your comments. I rechecked again and change to PLOS ONE requirement format for reference and citation.

**Reviewer 2 Comments**:

1. The theoretical background of the research is presented well - UTAUT2, perceived value theory and SOR theory are described alongside with scientific articles using them. Regarding the perceived value theory, the authors state: As a result, factors such as performance expectancy, effort expectancy, social influence, hedonic motivation, and habits are expected to contribute to viewers' perceived value of video streaming platforms, thereby influencing their subscription intentions (14-16). Thus, habits are considered a factor for influencing viewers' perceived value of video streaming platforms.
   Within 2.2. Mediating Role of perceived value, the authors state: Drawing from the S-O-R (Stimulus-Organism-Response) model (Mehrabian & Russell, 1974), which suggests that external stimuli trigger cognitive or emotional responses that lead to behavioral changes, this study explores how platform features and factors such as performance expectancy, effort expectancy, social influence, hedonic motivation, and habit (i.e., Stimulus) influence users' perceived value (i.e., Organism), subsequently impacting their subscription intentions (i.e., Response) on video streaming platforms (Jacoby, 28, 2002). Despite the theoretical foundation provided by the S-O-R model...
   Thus, once again, the authors consider habit as an influence factor of perceived value.
   ...but there is no research hypothesis regarding the mediation role of perceived value for the habit - subscription intention relationship.
   Maybe the authors should argument why this hypothesis was not included (although, as they mentioned previously, ... and habit (i.e., Stimulus) influence users' perceived value (i.e., Organism)..)

Author action: Thanks for your valuable comments. I gave some parts of explaination about this comments. First, this research found that perceived value mediates the relationship between performance expectancy and subscription intention of video streaming platforms (H6). These results are mostly consistent with other scholars’ findings (Xie et al., 2021; Yan et al., 2021; Fatima et al., 2021; Shaw & Sergueeva, 2019; Shoheib & Abu-Shanab, 2022). Secondly, this study found that perceived value mediates the relationship between effort expectancy and subscription intention of video streaming platforms (H7). The results of this study are mostly consistent with other scholars’ findings (Xie et al., 2021; Yan et al., 2021; Fatima et al., 2021). Thirdly, this research found that perceived value mediates the relationship between social influence and subscription intention of video streaming platforms (H8). The results of this study are mostly consistent with other scholars’ findings (Altalhi, 2020; Persaud & Schillo, 2017; Shaw & Sergueeva, 2019; Zia et al., 2022). Fourthly, this research found that perceived value mediates the relationship between hedonic motivation and subscription intention of video streaming platforms (H9). This is mostly consistent with other scholars’ findings (Fatima et al., 2021; Shaw & Sergueeva, 2019; Lapa, 2018; Ashraf et al., 2018). However, these is no such literature supporting for the mediation effect of perceived value between habit and subscription intention. Thus, this research will not add this hypothesis to investigate further.

To address the comments, I revised mentioned parts of contents in the introduction section, paragraph 4 line 12-15, stated as : “As a result, factors such as performance expectancy, effort expectancy, social influence and hedonic motivation are expected to contribute to viewers' perceived value of video streaming platforms, thereby influencing their subscription intentions.”

And to address the comments, I revised mentioned parts of contents in the theoretical background section, 2.2 Mediating role of perceived value, pharagraph 4, line 31-35, stated as: “ Drawing from the S-O-R (Stimulus-Organism-Response) model [54], which suggests that external stimuli trigger cognitive or emotional responses that lead to behavioral changes, this study explores how platform features and factors such as performance expectancy, effort expectancy, social influence and hedonic motivation (i.e., Stimulus) influence users' perceived value (i.e., Organism), subsequently impacting their subscription intentions (i.e., Response) on video streaming platforms.”

2. Both measurement and structural equation model should be statistically tested through tests/indicators such as: ChiSquare, RMSEA, PCLOSE and/or NFI,RFI,IFI,CFI and/or GFO,AGFI.

Author action: Thanks for your valuable comments. Because we are not doing covariance SEM, and we are doing PLS-SEM, So the covariance matrices are not what the model build on. In addition, some parts of mentioned fit measures are not great for SEM PLS because it’s more about making predictions than explaining things, and they are for CB SEM. So, an assessment of model fit of PLS-SEM is not include RMSEA, PCLOSE and/or RFI,IFI,CFI and/or GFO,AGFI, ChiSquare. However, in PLS SEM of model fit, mostly we consider about SRMR and NFI. Thus, I report the result of them in section of Methodology, data analysis, line 18-20, which is indicated as “In addition, in the model fit, the value for SRMR less than 0.08 and NFI above 0.9 are considered a good fit [33]. And in this study, the result of SRMR and NFI are 0.035 and 0.888, which indicated a good model fit (Table 2).” Furthermore, the table of model fit from PLS SEM is added in part of Methodology, data analysis, line 24, table 2.

3. Table 5 - Colliniarity Assessment has missing values. The authirs should explain in more detail what this table is about - I can only deduce (maybe wrongly) that the main diagonal elements are SQRAVE values and the other elements are partial correlation coefficient values.

Author action: Thanks for your valuable comments. Table 5 is wrongly reported and some parts of the values are missing in Colliniarity assessment. I reported again in section of Result, table 5, which indicated as
