# Supplementary material for: Explaining subscription intention for video streaming platforms in China: Integrating the UTAUT2 model, perceived value theory, and S-O-R theory
Source: PLoS One. 2025 May 8;20(5):e0322860. doi: 10.1371/journal.pone.0322860 (PMC12061175; doi:10.1371/journal.pone.0322860)
Supplement: Attachment — Submitted filename: Response_to_Reviewers_auresp_2.docx [file pone.0322860.s005.docx]

**Editor’s comments :**
Please review your reference list to ensure that it is complete and correct. If you have cited papers that have been retracted, please include the rationale for doing so in the manuscript text, or remove these references and replace them with relevant current references. Any changes to the reference list should be mentioned in the rebuttal letter that accompanies your revised manuscript. If you need to cite a retracted article, indicate the article’s retracted status in the References list and also include a citation and full reference for the retraction notice.

**Authors’ action:**

Thanks for your comments. Based on your suggestion, I rechecked the whole reference list. We deleted the duplicated references and replaced the retracted reference with new reference in the reference list. The reference of “Yoelianto, F., & Utami Tjhin, V. (2022). Social Isolation, A New Variable Affecting Behavioral Intention To Use Subscription Video On Demand. Journal of Theoretical and Applied Information Technology, 100(11),pp.3788-3797. ISSN: 1992-8645.” is retracted reference, which is replaced by the reference (line 8, page .7) of “Tsai, L. L. (2023). A deeper understanding of switching intention and the perceptions of non-subscribers. Information Technology & People, 36(2), 785-807.” In addition, the reference of “22. Endata.(2022). www.endata.com.cn.Endata - Data Intelligence Service Provider_Annual Box Office. [online].2022.<https://www.endata.com.cn/BoxOffice/BO/Year/index.html.>” is retracted reference, which is replaced by the reference (line 14, page .7) of “22. Huang, Y., Lv, Z., & Sui, Z. (2021, October). Where Should Existing Video Streaming Platforms Improve: A Comparative Analysis of Netflix and IQiyi. In 2021 International Conference on Public Relations and Social Sciences (ICPRSS 2021) (pp. 585-592). Atlantis Press.” Furthermore, the reference of “Ryu, K., & Jang, S. (2008). RETRACTED ARTICLE: Influence of restaurants' physical environments on emotion and behavioral intention. The Service Industries Journal, 28(8), 1151-1165.” is retracted reference, which is replaced by the reference (line 1, page.8) of “Zhang, H., Zheng, S., & Zhu, P. (2024). Why are Indonesian consumers buying on live streaming platforms? Research on consumer perceived value theory. Heliyon, 10(13).”

**Review 1 Comments:**

1. Upon thorough review of the manuscript titled "Explaining subscription intention for video streaming platforms in China: Integrating the UTAUT2 model, perceived value theory, and S-O-R theory," it has been observed that while the paper attempts to build upon an integrated theoretical framework, the linkage and interplay between the UTAUT2 model, perceived value theory, and S-O-R theory require further clarification. The rationale for the theoretical integration lacks sufficient depth to illustrate how these theories collectively contribute to a more comprehensive understanding of subscription intentions within the Chinese market.

**Authors’ action:**

Thanks for your comments. Based on your comments, we added contents to further clarification of the linkage and interplay between the UTAUT2 model, perceived value theory, and S-O-R theory in page 4, line 21-29, section of 1. introduction, which indicated as “In UTAUT2 model highlights the factors of performance expectancy, effort expectancy, social influence, hedonic motivation, and habit in shaping users' subscription intentions. In addition, the multi-dimensional aspects of perceived value are used in this research: emotional value, quality value, monetary value, and convenience value by perceivd value theory. Furthermore, based on S-O-R model, this study explores how platform features and factors such as performance expectancy, effort expectancy, social influence and hedonic motivation (i.e., Stimulus) influence users' perceived value (i.e., Organism), subsequently impacting their subscription intentions (i.e., Response) on video streaming platforms. Thus, this research integrates UTAUT2, perceived value theory, and S-O-R model to explain the mechanisms informing subscription intention.” In addition, based on your comments, we added contents to further illustrate how these theories collectively contribute to a more comprehensive understanding of subscription intentions within the Chinese market in page 4, line 40-43; page 5, line 1-5, section of 1. Introduction, which indicated as “Furthermore, new technologies, such as SVOD and artificial intelligence, have changed consumer behavior toward subscription packages in China. SVOD is related to the performance expectancy, effort expectancy, social influence, hedonic motivation, and habit that make consumers to increase their consumption of these platforms as access is unlimited. In addition, consumers in China use these platforms based on their preferences and choices that will help explain their goals. New customers enter these platforms on the basis that they feel attracted to the video content and perceive value during the trial period of the video streaming platform. Thus, this article integrates the UTAUT2 model, perceived value theory, and S-O-R theory to further clarify and contribute to reasons for the unstable subscription numbers of viewers in China. ”

2. Additionally, the operational definitions of key constructs, specifically 'perceived value' and 'attractiveness of alternatives,' may not be adequately tailored to the Chinese cultural context, which is vital for ensuring the relevance and applicability of the findings.
   **Authors’ action:**

Thanks for your comments. Based on your comments, we added the operational definitions of key construct of “perceived value” to adequately tailored to the Chinese cultural context, in page 7, line 25-30, section of 2.2 Mediating role of perceived value, 2. Theoretical background, which indicated as “And regarding to video streaming platforms in China, viewers’ perceived value is very important, which refers to the viewers' comprehensive evaluation of the services, content, experience and other aspects provided by the video streaming platform [49]. For example, in Tencent Video, Iqiyi, Youku and other mainstream video streaming platforms in China, they have continued to enhance the viewers’ perceived value by providing high-quality content, optimising the user experience, strengthening their branding, formulating a reasonable pricing strategy, and improving the quality of their services [50].”

And based on your comments, we added the operational definitions of key construct of “attractiveness of alternatives” to adequately tailored to the Chinese cultural context, in page 8, line 38; page 9, line 1-6, section of 2.3 Moderating role of attractiveness of alternatives, 2. Theoretical background, which indicated as “Video streaming market in China has formed a diversified competitive landscape, including the co-existence of short-form, long-form and live streaming [61]. In addition, the competition among video streaming platforms in China is extremely fierce, and platforms need to continuously innovate and optimize in terms of content quality, user experience, technological innovation and business models to meet the challenges of market competition. Thus, attractiveness of alternatives in video streaming platforms in China plays an important role in viewers’ subscription decisions [62].”

3. The sampling methodology employed raises concerns regarding potential biases and the generalizability of the study's conclusions. The combination of self-selection, volunteer, and convenience sampling methods, while pragmatic, may not provide a sample that is representative of the broader viewer population in China. Consequently, this could limit the external validity of the research.

**Authors’ actions:**

Thanks for your valuable comments. In this study, volunteer sampling refer to self-selection sampling techniques. Self selection is one of volunteer sampling technique, which is fully aware of all these questions, so we combine volunteer and self-selection to volunteer (self-selection). Because it is online questionnaire, convenience sampling method is suitable for access the online link of questionnaire. This study chooses these to sampling technique has been covered research question. We added the contents in section of page 10, line 15-21, which is indicated as “First of all, the sample size is large enough to cover the research question. Secondly, in the questionnaire, it has screening questions to filter out the issues of non-compliance. In addition, the age group is above 18, which can be covered. Furthermore, for viewers’ demographic, region and employment status, these two sampling technique has been assessed. Thus, in this study combination of volunteer (self-selection) and convenience sampling methods can be applied to this research. Furthermore, previous scholars are also adopt the similar sampling techniques in their studies [8, 15].” For more detailed revision, we revised the contents and added explanation of sampling methods in page 10, line 22-40, section of 3. Methodology, which indicated as “Volunteer sampling is the self-selection of sample members to participate in a study. Furthermore, self-selection sampling is when the study allows each case (usually an individual) to determine their willingness to participate in the study [70]. Self-selection cases are often selected because they have strong feelings or opinions about the research questions or stated goals. Therefore, the study publicized the need for cases through appropriate media advertising or by inviting volunteers to participate. Thus, it chose this sampling technique because some filtering questions (stated in the appendices of questionnaire) were applied to select the most appropriate samples from the target population in order to meet the researcher’s interests regarding the objectives of this study. Additionally, convenience sampling can be used when studying issues such as psychological mechanisms and processes where individual differences are small, the total population is unknown, or random sampling is difficult. This study investigates the influencing factors of subscription intention for VSPs in China, especially about the exploration of the psychological dimension of consumers. Furthermore, for larger survey samples, it is not easy to obtain a complete sample frame. Thus, this study chose convenience sampling to send the questionnaire link as a post through three major social media platforms to facilitate respondents to answer and submit the survey. For sample size, in this research, it uses G*Power (a power probability = 0.95, effect size = 0.15 (medium), alpha error probability = 0.05, number of predictors is 8, the minimum sample size for this study was determined to be 160 respondents [71]. Furthermore, according to Comrey and Lee (2013) conducted a study to assess the sufficiency of sample size for factor analysis, which concludes that an excellent factor analysis program would require a sample size of 1,000 while a sample size of 500 would be considered good [72].”

4.The adoption of measurement scales from various sources necessitates careful cultural adaptation and validation to ensure their appropriateness for the targeted demographic.

**Authors’ actions:**

Thanks for your valuable comments.

In this study, the measurement scale is adopted from previous studies. But, it is is reliable and validity through construct validity procedure. We added the contents in line 13-19, page 12, section of Methodology, which is indicated as “Additionally, the measurement scale is adopted from previous studies, which is considered valid indicated in Supporting Information_Appendix 1 Questionnaire [11, 21, 31, 46, 51, 60, 73, 74]. In this study, we did construct validity through 2 marketing experts from video stream industries, 3 university scholars, and 6 potential respondents (randomly selected) to ensure the clarity and comprehensibility of the questionnaire prior to pilot test. Then, this study proceed to pilot study. In addition, digital media consumption and subscription has become a global (e.g. Netflix, HBO Max, IQIYI) tread that is more familiar with most consumers.”

5. The data analysis could also benefit from a more in-depth approach, including the exploration of potential differences across demographic groups through multi-group analysis, which the study has not fully leveraged. These aspects are critical for enhancing the robustness and integrity of the study, and addressing them would significantly strengthen the paper's contributions to the field.

**Authors’ actions:**

Thanks for your valuable comments. We run the multi-group analysis with female and male. And I added the section of Multi-group analysis in line 6-16, page 7, section of 4. Results, which is indicated as “In the final part of the research, we evaluated the significant differences between female and male that existed in the effects of influencing factors on subscription intention (S). In the multi-group comparison, gender had a significant difference in the moderation impact of AOA on the relationship between perceived value (PV) and subscription (S). In addition, the differences in path coefficient of moderation (AoA*PV) revealed that female group has relatively greater impact in comparison to male group (path diff = 0.135, p = 0.023). All the other differences in the hypothesized relationships were found insignificant. The results of the multi-group analysis are summarized in table 10.

**Reviewer #3 Comments:**

1. Introduction
   It would be good to indicate why factors such as performance expectancy, effort expectancy, social influence and hedonic motivation are selected to determine intention for video streaming platforms? why UTAUT2 is selected and not other version of technology model such as UTAUT, TAM3 etc. Why SOR and perceived value theory is included? Also, why attractiveness of alternatives is included? It should include some discussion on the context that could strengthen or weaken the relationship.

**Authors’ actions:**

Thanks for your valuable comments. Based on your suggestion, we added the reason why factors such as performance expectancy, effort expectancy, social influence, habit and hedonic motivation are selected to determine subscription intention for video streaming platforms, in page 3, line 1-5, section of 1. Introduction, which is indicated as “SVOD is related to the performance expectancy, effort expectancy, social influence, hedonic motivation, and habit that make consumers increase their consumption of these platforms as access is unlimited. According to literature, performance expectancy, effort expectancy, social influence, hedonic motivation, and habit are based on the Extended Unified Theory of Technology Acceptance and Use (UTAUT2) [11]. ” And based on your comments, we added reasons why UTAUT2 is selected and not other version of technology model such as UTAUT, TAM etc in line 5-14, page 3, section of 1. Introduction, which is indicated as “SVOD is related to the performance expectancy, effort expectancy, social influence, hedonic motivation, and habit that make consumers increase their consumption of these platforms as access is unlimited. According to literature, performance expectancy, effort expectancy, social influence, hedonic motivation, and habit are based on the Extended Unified Theory of Technology Acceptance and Use (UTAUT2) [11]. ” In addition, based on your comments, we added reasons why SOR and perceived value theory is included in line 28-43, page 3; line 1-7, page 4, section of 1. Introduction, which is indicated as “Overall, it is important to look into different aspects of how value is perceived. First, the Theory of Consumer Value (TCV) provides a clear delineation of consumption value that helps determine consumers’ decision-making intentions [20]. Understanding this delineation is important for video streaming platform providers as they can benefit from understanding why individuals use video streaming platforms and why they choose to watch certain content. In addition, studying the value of consumption can also provide users’ service providers with more interactive and engaging content to attract new users and retain existing users [17]. Many studies within the literature use perceived value theory for behavioral intention, especially purchase intention. But most research only looks at how different aspects of how consumers’ perceived value affects their purchase decision [21]. Although only the perceived value theory bridges the gap between in-depth research on multi-dimensional perceived value and the impact of perceived value on consumer behavior, there is still a lack of research on the antecedents of perceived value. The effect of performance expectancy, effort expectancy, social influence, hedonic motivation, and habit on subscription intention have not been addressed by using perceived value theory, which is a research gap. In addition, it is a research gap as to whether perceived value has a mediating effect between performance expectancy and subscription intention, effort expectancy and subscription intention, social influence and subscription intention, and hedonic motivation and subscription intention, which needs more research theories to resolve. Furthermore, researchers have recently proposed an integrated S-O-R framework that includes cognitive and emotional systems and all long-term memories from the past [22]. Different scholars expand the S-O-R model by multiple factors in stimulus, organism, and response. Based on the above critical arguments, this research will expand the S-O-R model to apply technology acceptance variables to internal stimuli, i.e., performance expectancy, effort expectancy, social influence, hedonic motivation for viewers. And this research will also expand the organism of S-O-R to perceived value and the response of S-O-R to subscription intention.” Moreover, based on your suggestion, we added reasons why attractiveness of alternatives is included in line 8-11, page 4, section of 1. Introduction, which is indicated as “There is a lot of competition among video streaming platforms. In addition, customer perceptions of the attractiveness of alternatives may be an important factor in attracting customers away from their current service provider [23]. Thus, the moderator of attractiveness of alternative will reflect the real world of the video streaming industry.”

2. Theoretical background
a. Only UTAUT2 is discussed, how about perceived value theory and SOR?

**Authors’ actions:**

Thanks for your valuable comments. Perceived value theory and SOR is discussed in line 1-13, page 8, 2.2 Mediating role of perceived value, section of 2. Theoretical background, which is indicated as “This study introduces perceived value as the mediator between the driving factors and subscription intention on the basis of the Perceived Value Theory [51] and S-O-R (Stimulus-Organism-Response) model [52]. Perceived Value Theory posits that consumer behavior is influenced by their assessment of the benefits and costs associated with a product or service. In the context of video streaming platforms, consumer-perceived value refers to users' evaluations of the benefits and costs of different platforms, serving as a link between the value proposition offered and users' purchasing behavior [51].

Drawing from the S-O-R (Stimulus-Organism-Response) model [52], which suggests that external stimuli trigger cognitive or emotional responses that lead to behavioral changes, this study explores how platform features and factors such as performance expectancy, effort expectancy, social influence and hedonic motivation (i.e., Stimulus) influence users' perceived value (i.e., Organism), subsequently impacting their subscription intentions (i.e., Response) on video streaming platforms [22]. Despite the theoretical foundation provided by the S-O-R model, there is limited research utilizing it to investigate factors influencing subscription intentions in video streaming platforms.”.

b. For the proposed hypotheses, the relationship is positive or negative? It is not indicated in the H1 to H5.

**Authors’ actions:**

Thanks for your valuable comments. Based on your comments, we revised the H1 to H5 accordingly, in line 10, 21, 29, page 6; line 4, 15, page 7, section of 2.1 Unified theory of acceptance and use of technology (UTAUT2) model, 2. Theoretical background, which is indicated as

“H1 Performance expectancy positively affects the subscription intention of video streaming platforms.

H2 Effort expectancy positively affects the subscription intention of video streaming platforms.

H3 Social influence positively affects the subscription intention of video streaming platforms.

H4 Hedonic motivation positively affects the subscription intention of video streaming platforms.

H5 Habit positively affects the subscription intention of video streaming platforms.”

c. Habit is not tested for mediation? The research model is not able to be detected in the manuscript.

**Authors’ actions:**
Thanks for your valuable comments. However, based on UTAUT2 , habit positive influence subscription intention. In addition, based on literature, there is non robust evidence for perceived value mediates the relationship between habit and subscription intention. Thus, in this research model, we did not test the mediation effect of perceived value between habit and subscription intention. Some scholars found perceived value positively affect habit (Zhong & Moon, 2022; Lin et al., 2018; Fatima et al.,2021). And some scholars found habit is positively affect subscription intention (ALKAWSI et al.,2020; Dhiman, 2019). However, there is no support in literature for the mediation of habit between perceived value and subscription intention. Furthermore, when consumers form habit, they will subscript directly, no matter if there are mediators.

3. Methodology
   a. as it is a combination of self-selection, volunteer, and convenience sampling methods are used for the selection of respondents, it would be good to explain each of the method and how do all these techniques contribute to 506 respondents?

**Authors’ actions:**

Thanks for your valuable comments. Based on your comments, we revised the the sampling technique to volunteer (self-selection). For more detailed revision, we added the contents and explanations in line 19-29, paragraph 1, section of 3. Methodology, which indicated as “ Volunteer sampling is the self-selection of sample members to participate in a study. Furthermore, self-selection sampling is when the study allows each case (usually an individual) to determine their willingness to participate in the study [70]. Self-selection cases are often selected because they have strong feelings or opinions about the research questions or stated goals. Therefore, the study publicized the need for cases through appropriate media advertising or by inviting volunteers to participate. Thus, it chose this sampling technique because some filtering questions (stated in the appendices of questionnaire) were applied to select the most appropriate samples from the target population in order to meet the researcher’s interests regarding the objectives of this study. Additionally, convenience sampling can be used when studying issues such as psychological mechanisms and processes where individual differences are small, the total population is unknown, or random sampling is difficult. This study investigates the influencing factors of subscription intention for VSPs in China, especially about the exploration of the psychological dimension of consumers. Furthermore, for larger survey samples, it is not easy to obtain a complete sample frame. Thus, this study chose convenience sampling to send the questionnaire link as a post through three major social media platforms to facilitate respondents to answer and submit the survey.”

b. How does sample size is determined? indicate the technique.

**Authors’ actions:**

Thanks for your valuable comments. Based on your comments, we added the the sample size determination, in line 35-40, page 10, section of 3. Methodology, which indicated as “For sample size, in this research, it uses G*Power (a power probability = 0.95, effect size = 0.15 (medium), alpha error probability = 0.05, number of predictors is 8, the minimum sample size for this study was determined to be 160 respondents [71]. Furthermore, according to Comrey and Lee (2013) conducted a study to assess the sufficiency of sample size for factor analysis, which concludes that an excellent factor analysis program would require a sample size of 1,000 while a sample size of 500 would be considered good [72].”

c. It would be good to include the measures/items for the constructs? also the source of the measures/items.

**Authors’ actions:**

Thanks for your valuable comments. Based on your comments, we added measurement scales/items in Supporting Information_Appendix 1 Questionnaire. And we added contents in line 14, page 12, section of Methodology, which is indicated as “Additionally, the measurement scale is adopted from previous studies, which is considered valid indicated [11, 21, 31, 46, 51, 60, 73, 74] in Supporting Information_Appendix 1 Questionnaire.”.

d. Did any pilot study is performed?

**Authors’ actions:**

Thanks for your valuable comments. This study did the construct validity and pilot study. And according to your comments, we added the contents of construct validity and pilot study in line 13-21, page 12, section of Methodology, which is indicated as ”Additionally, the measurement scale is adopted from previous studies, which is considered valid indicated [11, 21, 31, 46, 51, 60, 73, 74] in Supporting Information_Appendix 1 Questionnaire. In this study, we did construct validity through 2 marketing experts from video stream industries, 3 university scholars, and 6 potential respondents (randomly selected) to ensure the clarity and comprehensibility of the questionnaire prior to pilot test. Then, this study proceed to pilot study. In addition, digital media consumption and subscription has become a global (e.g. Netflix, HBO Max, IQIYI) tread that is more familiar with most consumers. And this study conducted a final pilot test of the questionnaire draft with 72 users of video streaming platforms in China to assess the construct items analysis, validity analysis, and reliability analysis [75], which indicated all results considered satisfactory.”

4. Results
In Table 7. Structural model assessment, the direct relationships are presented but is not tested as Hypotheses.

**Authors’ actions:**

Thanks for your valuable comments. In this study, the research model is to test the mediation effect of perceived value between performance expectancy and subscription intention, effort expectancy and subscription intention, social influence and subscription intention, hedonic motivation and subscription intention. And the total direct effect is to assess the relationship between performance expectancy and subscription intention, effort expectancy and subscription intention, social influence and subscription intention, hedonic motivation and subscription intention. Thus, even though this study showed the direct effect of

Performance expectancy → Perceived value

Effort expectancy → Perceived value

Social influence → Perceived value

Hedonic motivation → Perceived value

But it is not the hypothesis for this study. The direct effect is supplement for mediation effect, which is significant. Thus, it can be concludes the mediation effect is partial mediation, not full mediation. Within all the mediation effect, the mediation effect of perceived value between effort expectancy and subscription intention is the most significant. And the mediation effect of perceived value between Social influence and subscription intention is less significant. Based on your suggestion, we revised the table 7 to clearly indicated the result. And we deleted “the direct effects of Performance expectancy → Perceived value, Effort expectancy → Perceived value, Social influence → Perceived value, Hedonic motivation → Perceived value, Perceived value → Subscription intention”. Because it is duplicated with total mediation effect. Thus, we revised table 7 in page 19, section of 4. Results, which is indicated as below.
